# Getting the Most Out of Your Genome-Wide Association Study Using Polygenic Risk Scores

**DOI:** 10.1128/jcm.00223-23

**Published:** 2023-06-05

**Authors:** Emily Maggioncalda, Evan Snitkin

**Affiliations:** a Department of Microbiology and Immunology, University of Michigan Medical School, Ann Arbor, Michigan, USA; National Institute of Allergy and Infectious Diseases

## Abstract

Determining the risk of a phenotypic outcome is a complex balance of variants “for” or “against” the phenotype, which in the context of human genetic diseases have been summarized using polygenic risk scores. In a previously published article (K. T. Hellmann, L. Challagundla, B. M. Gray, D. A. Robinson, J Clin Microbiol 61:e01412-22, 2023, https://doi.org/10.1128/jcm.01412-22), Hellmann and colleagues demonstrate how the use of a bacterial polygenic risk score to predict S. epidermidis infection versus colonization in neonates led to both increases in predictive accuracy and improved generalizability to external data.

## TEXT

The expanded accessibility of whole-genome sequencing has opened the door to understanding complex phenotypes that have eluded placement into the “mutation in *yfg* leads to phenotype Y” category. Although there are examples of single genes that can result in a change of phenotype in both human genetics (cystic fibrosis and mutations in the cystic fibrosis transmembrane conductance regulator [*CFTR*] gene [1]) and microbial genetics (Mycobacterium tuberculosis rifampicin resistance and RNA polymerase [*rpoB*] mutations [2]), phenotypes are often more complex.

Genome-wide association studies (GWAS) have become an integral tool for identifying complex genetic variation associated with clinically important phenotypes in a range of organisms. Although GWAS methodologies in the context of human studies are quite mature, there are significant differences in bacterial evolution that have prevented the direct application of these methods. The most significant challenge for executing bacterial GWAS (bGWAS) is the lack of systematic recombination in the evolution of most bacterial species. This lack of recombination has the potential to create linkage between variants that can make it challenging to distinguish variants causal of phenotypes versus passenger variants that are merely associated. In the most extreme cases, you can have what is called lineage effects, wherein a phenotype is either present or absent in all members of a bacterial lineage, which makes it essentially impossible to employ bGWAS to identify causal variants, due to existence of large numbers of variants shared between all members of a lineage. To get around these lineage effects, researchers have taken advantage of the fact that for many phenotypes of clinical significance (e.g., antibiotic resistance and growth in host environments), there is sufficiently strong selective pressure for the phenotype that they emerge multiple times within and across lineages. This observation of repeated emergence (i.e., convergence) of phenotypes has enabled the development of bacterial-specific methods that specifically seek variants that co-converge with phenotypes of interest more than would be expected by chance.

In some instances, genome-wide association studies can identify putative high-impact variants associated with a phenotype (3), but more often than not, not all cases can be explained by high-impact variants alone and/or there is not enough power to confidently identify variants associated with the given phenotype (4, 5). Polygenic risk scores aim to fully utilize the information in the genome by not only considering variants of high impact, but also including low impact variants (6, 7). Hellmann and colleagues sought to employ this approach in the context of bacteria by using machine learning methods to generate a polygenic risk score for S. epidermidis bacteremia versus contamination of blood cultures in NICU neonates. To do this, they utilized accessory genome (AG) content of isolates, as well as single nucleotide polymorphisms (SNPs) in comparison to a reference genome. Initial bGWAS provided single locus risk scores of the different AG and SNP variants in the form of odds ratios. The polygenic risk score for each isolate was then determined based on the sum of the effect sizes of variants present in the isolate, with decisions on which variants to include as part of the score made based on significance thresholds of individual variants, and removal of redundant variants due to linkage disequilibrium. The isolate’s polygenic risk score and the outcome of the patient it was isolated from (bacteremia or presumed contamination) was then fed into a logistic regression model to determine how well the polygenic risk score could predict which bin the sample fell into. As a result of this, the authors were able to identify a model that provided increased accuracy in determining infection or contamination compared to other previously published methods utilizing more traditional models limited to a smaller number of known virulence-associated loci.

The inability to maintain predictive accuracy of machine learning models when applied to external test data is a well-established concern when applying these models to health care decision making (8). To assess the generalizability of their model, the authors applied it to data sets not used in the model training process. This involves calculating the polygenic risk scores for these out-sample isolates and using the previously developed logistic regression model to determine sensitivity and specificity of binning the predicted outcomes. It is in the evaluation of these out-sample data sets that the strength of the polygenic risk score is demonstrated. Although there is a general decrease in model accuracy on the novel data sets compared to the initial training data, it is much improved over the performance of the comparator traditional models. The decreased reliance on a limited number of high-impact variants allows for increased flexibility in the determination of isolate outcome. This may be compared to “seeing the forest for the trees”, a model that uses a small number of high-impact variants may miss something in the larger “ecosystem” of variants that the polygenic score model is able to grasp.

Despite these promising results, the clinical application of polygenic risk scores in the context of infectious diseases requires careful consideration (6–8). In genetic counseling settings, a patient may be provided with a risk score for certain cancers, diabetes, Alzheimers, etc. (7) and can then choose to follow up on that information with actions to help decrease their risk in the long term. If a patient starts to experience signs or symptoms of the disorder, a clinician can then address it. The balance of risk/benefit of preventative management in these settings is much lower compared to the setting of a positive blood culture. The time to treatment is of critical importance to neonatal outcomes (9), and a “watch and wait” approach has an inherent higher risk in this scenario.

In addition, as previously mentioned, many of the limitations with machine learning models are based on the training of the initial model. A model such as the one used here is limited in its knowledge to only variants it has seen before in order to calculate a polygenic risk score and make a prediction. This leaves a blindspot of novel variants being missed as “of increased risk” if they are completely absent from the data the model was trained on. This issue is addressed in the authors’ discussion of performance limitations on the novel data sets based on similarity to the training data. The integration of additional factors external to the sampled strain’s variants, such as relevant patient factors (degree of prematurity and underlying health factors) and broader information on strain biology (rate of horizontal gene transfer and evolutionary rate), could help provide additional model context in situations where (i) patient state is a significant factor in outcome of colonization versus infection and (ii) the strain contains variants not present in prior model training. This second case could also be addressed by having an identity filtering step prior to model input to determine if the strain is represented in the training data. In order to integrate this into the clinical microbiology setting, these factors would need to be established to make this method widely applicable across institutions.

In conclusion, the authors’ use of a polygenic risk score for assignment of S. epidermidis as contamination versus bacteremia is a positive test case for a methodology already utilized in human genetics. This method shows promise for applications in other infectious disease settings and could be a valuable decision making tool provided that the limitations of the models are well-established up front.
